# γ-H2AX/53BP1/pKAP-1 foci and their linear tracks induced by *in vitro* exposure to radon and its progeny in human peripheral blood lymphocytes

**DOI:** 10.1038/srep38295

**Published:** 2016-12-06

**Authors:** Defang Ding, Yaping Zhang, Jing Wang, Xufei Wang, Dunhuang Fan, Linfeng He, Xuxia Zhang, Yun Gao, Qiang Li, Honghong Chen

**Affiliations:** 1 Department of Radiation Biology, Institute of Radiation Medicine, Fudan University, Shanghai, 200032, China; 2 Department of Radiology, Shanghai Jiao Tong University Affiliated Sixth People’s Hospital, Shanghai, 200233, China; 3 Institute of Modern Physics, Department of Nuclear Science and Technology, Fudan University, Shanghai, 200433, China; 4 Department of Radiological Health, Institute of Radiation Medicine, Fudan University, Shanghai, 200032, China; 5 Division of Ionizing Radiation Measurement Technology, Shanghai Institute of Measurement and Testing Technology, Shanghai, 201203, China

## Abstract

The biodosimetric information is critical for evaluating the human health hazards caused by radon and its progeny. Here, we demonstrated that the formation of phosphorylated histone variant H2AX (γ-H2AX), p53-binding protein 1 (53BP1) and phosphorylated KRAB-associated protein 1 (pKAP-1) foci and their linear tracks in human peripheral blood lymphocytes (HPBLs) *in vitro* exposed to radon and its progeny were dependent on the cumulative absorbed dose of radon exposure but was unrelated to the concentration of radon. Among them, γ-H2AX foci and its linear tracks were the most sensitive indicators with the lowest estimable cumulative absorbed dose of 1.74 mGy from their linear dose-response curves and sustained for 12 h after termination of radon exposure. In addition, three types of foci showed an overdispersed non-Poisson distribution in HPBLs. The ratios of pKAP-1/γ-H2AX foci co-localization, 53BP1/γ-H2AX foci co-localization and 53BP1/pKAP-1 foci co-localization were significantly increased in HPBLs exposed to radon while they were unrelated to the cumulative dose of radon exposure, suggesting that γ-H2AX, pKAP-1 and 53BP1 play an important role in the repair of heterochromatic double-strand breaks. Altogether, our findings provide an experimental basis for estimating the biological dose of internal α-particle irradiation from radon and its progeny exposure in humans.

The human health risk associated with environmental radon exposure including occupational, indoor or drinking water consumption exposure to high radon and its progeny has been recognized for decades. Radon and its progeny have been classified as a category 1 human carcinogen by the International Agency for Research on Cancer^1^. After inhalation or ingestion, radon and its progeny are deposited in lungs or bone marrow adipose and bone tissues, produce high linear energy transfer (LET) α-particle irradiation on the respiratory cells and bone marrow hematopoietic stem/progenitor cells^2^,^3^,^4^, and responsible for the lung cancer and leukemia risk^5^,^6^,^7^,^8^,^9^,^10^. The α-particle exposure is the major type of radiation generated by inhaled or ingested radon and its progeny, although some β particles and γ-rays are also emitted. It is well established that biodosimetric information is critical for evaluating the health hazards caused by ionizing radiation (IR). However, exposure to radon is a type of chronic, cumulative, low-dose and non-uniform radiation exposure to human body, and a simple and reliable method for estimating the biological dose from internal α-particle irradiation is still unavailable.

The high LET α-particle irradiation has been demonstrated to induce clustered double-strand break (DSB) damage in DNA, a type of damage that is more difficult to repair than the isolated DSBs induced by low-LET radiation^11^,^12^. Recent studies have shown that clustered DSBs can be repaired with slow kinetics via the microhomology-mediated non-homologous end joining (NHEJ) pathway in the G1 phase of the cell cycle^13^, and that heterochromatic DSB repair is a critical rate-limiting step in the repair process^13^. In the DNA damage response (DDR), phosphorylated histone variant H2AX (γ-H2AX), a molecular marker for DSBs, provides a platform for the accumulation of DSB repair factors in mammalian cells^11^. p53-binding protein 1 (53BP1), a transducer in DDR, not only has a critical decision-making role in choosing DSB repair pathways but also promotes heterochromatic DSB repair in G1 phase cells by recruiting the phosphorylated ataxia telangiectasia mutated protein (p-ATM)^14^,^15^. KRAB-associated protein 1 (KAP-1), a molecular marker for heterochromatin, plays an important role in the chromatin remodeling process. In cells that have not been subjected to stress injury, KAP-1 exists in dense heterochromatin in a SUMOylated form. When DSBs occur in heterochromatin, 53BP1-dependent concentrated p-ATM phosphorylate KAP-1 and induce the formation of phosphorylated KAP-1 (pKAP-1) foci, a molecular marker for heterochromatic DSBs, leading to local relaxation of heterochromatin structure at the sites of DSBs. As a result, repair proteins can readily access the damaged sites to carry out repair^16^.

Immunofluorescence examination of γ-H2AX foci in peripheral blood lymphocytes (PBLs) has been used to estimate the biological dose from uniform external radiation^17^,^18^,^19^,^20^,^21^,^22^,^23^ and local irradiation^24^,^25^. Compared to the dicentric assay as the “gold standard” for biodosimetry analysis, γ-H2AX foci approach is a simple and rapid assay with a high sensitivity as low as several mGy^17^,^26^. Some laboratories have developed methods for automatic detection of γ-H2AX foci that permit high-throughput screening of populations^27^,^28^,^29^,^30^. Our recent study has established a γ-H2AX foci-based assay to determine biological dose to red bone marrow (RBM) in radon-inhaled rats, in which the linear γ-H2AX foci track in rat PBLs and bone-marrow lymphocytes (BMLs) can serve as a biomarker to determine whether the body is suffered from high radon exposure in the absence of other internal radiation from α-particle emitting radionuclides and high-LET external irradiation^31^. γ-H2AX foci quantitation in rat PBLs can be used to estimate RBM-absorbed doses with the established linear dose-response curve of γ-H2AX foci after *in vitro* radon exposure and the established ratio of RBM- to PBLs-absorbed doses after *in vivo* radon exposure^31^. In addition, IR-induced 53BP1 foci is also a useful biomarker for detecting radiation exposure after radionuclide incorporation, even for absorbed doses to the blood below 20 mGy^32^. In the present study, we established dose-response curves for the linear γ-H2AX/pKAP-1 foci tracks and for individual γ-H2AX/pKAP-1 foci induced by *in vitro* exposure of human PBLs (HPBLs) to radon and its progeny. Moreover, we explored the dose-response relationships of the linear 53BP1 foci tracks and individual 53BP1 foci. We also examined the characteristics of the formation, elimination and distribution of the three types of foci, and the status of co-localization of each pair of the three types of foci in HPBLs exposed to radon and its progeny, and further determined the repair characteristics of the heterochromatic DSBs induced by internal α-particle irradiation from radon and its progeny exposure in HPBLs. The findings of this study provide an experimental basis for using the γ-H2AX foci-based assay to estimate the biological dose of internal α-particle irradiation from radon and its progeny exposure in humans.

## Results

### Exposure to radon and its progeny induces dose-dependent formation of linear γ-H2AX, 53BP1 and pKAP-1 foci tracks in HPBLs

**Figure 1:**
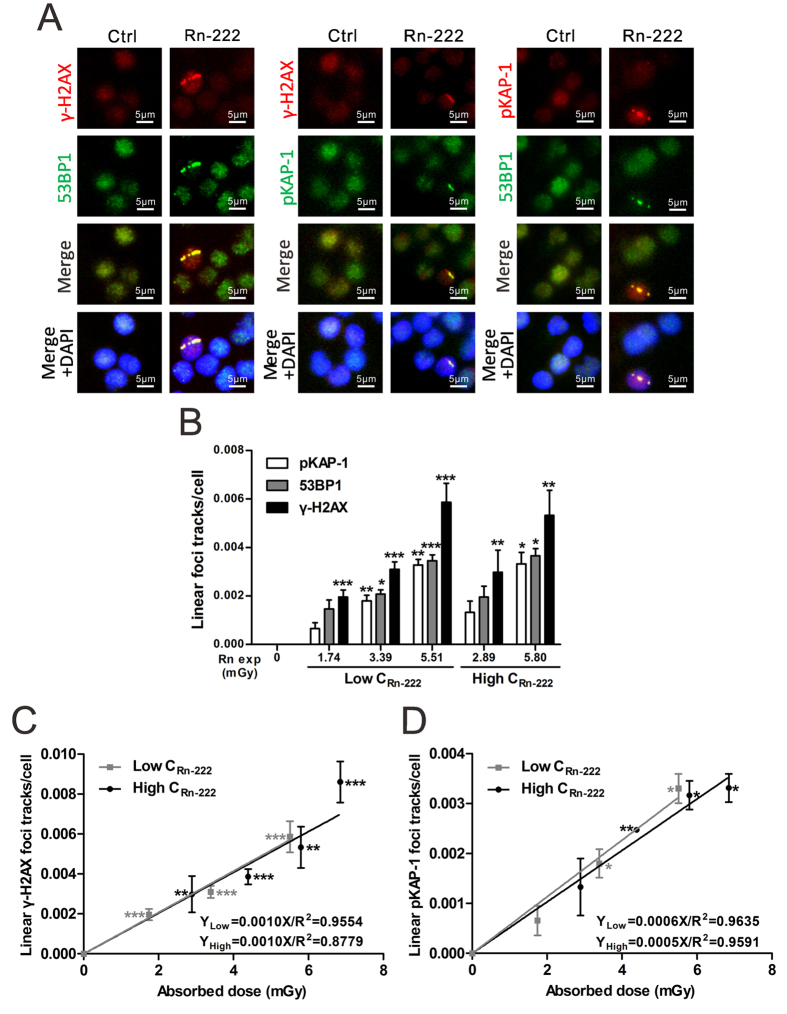
The formation of linear γ-H2AX, 53BP1 and pKAP-1 foci tracks in HPBLs *in vitro* exposed to radon and its progeny. (**A**) Representative images of linear γ-H2AX, 53BP1 and pKAP-1 foci tracks in HPBLs induced by *in vitro* exposure to radon and its progeny. (**B**–**D**) Dose-response relationship between the yields of linear γ-H2AX, 53BP1 and pKAP-1 foci tracks and cumulative radon exposure dose in HPBLs. Two to four thousand HPBLs from each sample were used for linear γ-H2AX, 53BP1 and pKAP-1 foci track quantitation. The data are presented as the mean ± standard deviation of three human blood samples. Red, γ-H2AX and pKAP-1; green, pKAP-1 and 53BP1; blue, DNA stained with DAPI. 1000 × magnification. *P < 0.05, **P < 0.01, ***P < 0.001 compared with the control group that was exposed to the natural background concentration of radon for the same protein. Rn exp, radon exposure.

As shown in Fig. 1A, *in vitro* radon exposure at 1.74–6.85 mGy induced not only the formation of linear γ-H2AX foci tracks but also the formation of linear tracks of 53BP1 and pKAP-1 foci in HPBLs. Moreover, some tracks were partial, which is the result of the α-particle range and spherical shape of HPBLs adhered to membrane. While the linear γ-H2AX foci tracks were consisted of continuous and intermittent linear tracks, the linear 53BP1/pKAP-1 foci tracks were mostly the intermittent linear tracks. The yields of these linear foci tracks were increased significantly in a dose-dependent manner (Fig. 1B). In contrast, no linear γ-H2AX, 53BP1 or pKAP-1 foci tracks were observed in HPBLs that received background levels of radon exposure. It is worth noting that at the same absorbed dose, the levels of linear γ-H2AX foci tracks were approximately 1.45–1.65 and 2.05–2.25 folds of the levels of linear 53BP1 foci and pKAP-1 foci tracks, respectively. The yield of linear γ-H2AX foci tracks was markedly increased over the background level when the absorbed dose from radon exposure was as low as 1.74 mGy, while the yields of linear pKAP-1 and 53BP1 foci tracks were significantly higher than the corresponding background values only with the cumulative dose of radon reaching 3.39 mGy (Fig. 1B). These results demonstrate that the formation of linear γ-H2AX foci tracks in HPBLs is more sensitive to radon exposure than the formation of the other two types of foci tracks.

Based on the above findings, a dose-response curve of the linear γ-H2AX foci tracks was constructed in HPBLs exposed to radon and its progeny (0–6.85 mGy) under two distinct exposure conditions: low concentration of radon at ~500 kBq/m^3^ and high concentration of radon at ~1000 kBq/m^3^ (Fig. 1C). A dose-response curve of the formation of linear pKAP-1 foci tracks was also established (Fig. 1D). The results show that both linear γ-H2AX foci tracks and linear pKAP-1 foci tracks exhibit a linear dose-response relationship with the absorbed dose from radon exposure. It is noteworthy that the dose-response curve of linear γ-H2AX foci tracks after exposure of HPBLs to radon with the high concentration overlaps with that obtained after exposure of HPBLs to radon with the low concentration. Similar results were obtained in dose-response curves of linear pKAP-1 foci track formation. These results indicate that the yields of linear γ-H2AX foci tracks and linear pKAP-1 foci tracks are determined by the cumulative absorbed dose from radon exposure but are independent on radon concentrations for exposure.

### Exposure to radon and its progeny induces dose-dependent formation of γ-H2AX, 53BP1 and pKAP-1 foci in HPBLs

**Figure 2:**
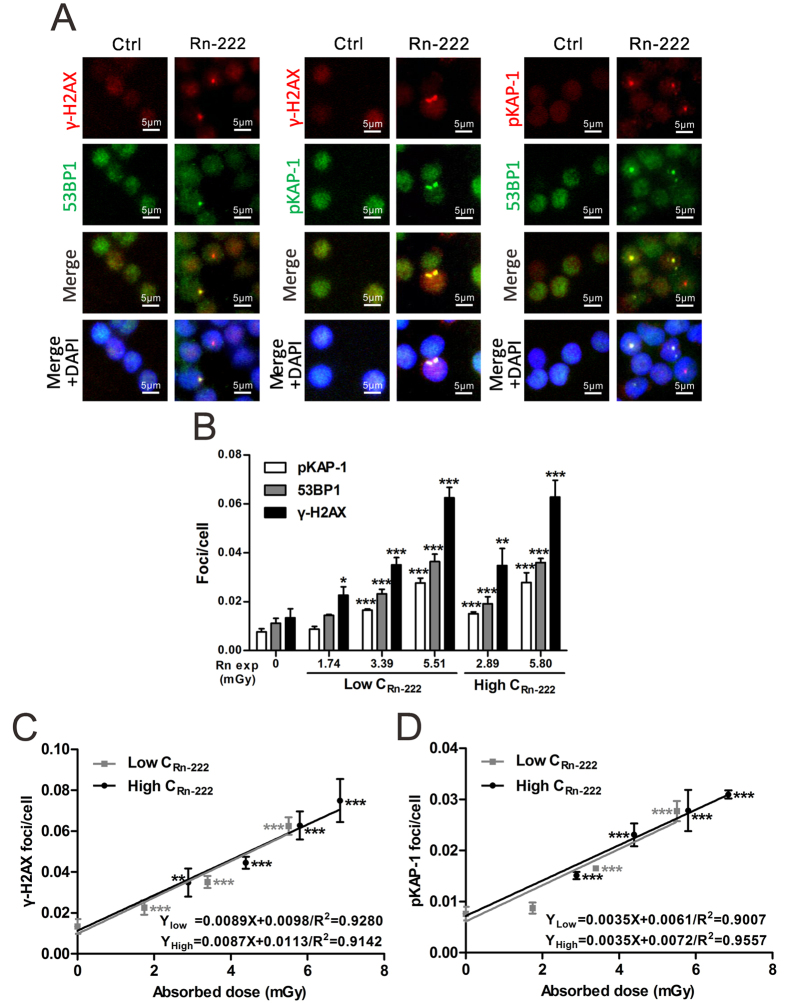
The formation of γ-H2AX, 53BP1 and pKAP-1 foci in HPBLs *in vitro* exposed to radon and its progeny. (**A**) Representative images of γ-H2AX, 53BP1 and pKAP-1 foci in HPBLs induced by *in vitro* exposure to radon and its progeny. (**B**–**D**) Dose-response relationship between the yields of γ-H2AX, 53BP1 and pKAP-1 foci and cumulative radon exposure dose in HPBLs. Two to four thousand lymphocytes from each sample were used for γ-H2AX, 53BP1 and pKAP-1 foci quantitation. The data are presented as the mean ± standard deviation of three human blood samples. Red, γ-H2AX and pKAP-1; green, pKAP-1 and 53BP1; blue, DNA stained with DAPI. 1000 × magnification. *P < 0.05, **P < 0.01, ***P < 0.001 compared with the control group that was exposed to the natural background concentration of radon for the same protein. Rn exp, radon exposure.

**Figure 3:**
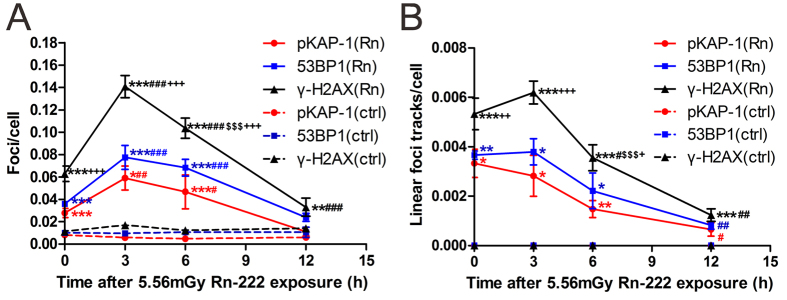
Persistent formation of γ-H2AX, 53BP1, pKAP-1 foci and their linear tracks in HPBLs after termination of exposure to radon and its progeny. (**A**) Temporal changes in the yields of γ-H2AX, 53BP1 and pKAP-1 foci in HPBLs immediately (0 h) after termination of radon exposure and at 3, 6 and 12 h after termination of radon exposure. (**B**) Temporal changes in the yields of linear γ-H2AX, 53BP1 and pKAP-1 foci tracks in HPBLs at 0, 3, 6 and 12 h after termination of radon exposure. Two to four thousand lymphocytes from each sample were used for γ-H2AX, 53BP1 and pKAP-1 foci and their linear tracks quantitation. The data are presented as the mean ± standard deviation of three human blood samples. For each protein: *P < 0.05, **P < 0.01 and ***P < 0.001 compared with the control group that was exposed to the natural background concentration of radon; ^#^P < 0.05, ^##^P < 0.01, ^###^P < 0.001 compared with 0 h after termination of radon exposure; ^$^P < 0.05, ^$$^P < 0.01, ^$$$^P < 0.001 compared with 3 h after termination of radon exposure. ^+^P < 0.05, ^++^P < 0.01, ^+++^P < 0.001 compared with 53BP1 and pKAP-1 foci or their linear tracks at corresponding time point after termination of radon exposure.

The formation of γ-H2AX, 53BP1 and pKAP-1 foci was observed at the levels of 0.0029–0.0198 foci/cell, 0.0015–0.0128 foci/cell and 0.0028–0.0095 foci/cell, respectively, in HPBLs after background radon exposure (Fig. 2A,B). There were no significant differences in the background foci levels observed at various time points during the course of 6–18 h *in vitro* HPBL culture (Fig. 3A). Similar results for the background level of γ-H2AX foci (0.0012–0.0176 foci/cell) and 53BP1 foci (0.0012–0.0333 foci/cell) in HPBLs have been reported^32^.

As shown in Fig. 2, radon exposure at 1.74–6.85 mGy induced the formation of γ-H2AX, 53BP1 and pKAP-1 foci in HPBLs (Fig. 2A), and the yields of these foci increased significantly as the cumulative absorbed dose increased (Fig. 2B). Like the yields of the three types of linear foci tracks, the yield of γ-H2AX foci was obviously higher than the yields of 53BP1 and pKAP-1 foci at the same absorbed dose. Specifically, the levels of γ-H2AX foci were 1.55–1.75 and 2.15–2.35 folds of the levels of 53BP1 foci and pKAP-1 foci, respectively. Moreover, at a cumulative dose of as low as 1.74 mGy from radon exposure, the yield of γ-H2AX foci was significantly higher than the background value, indicating that γ-H2AX foci shows a similar sensitivity as the linear γ-H2AX foci tracks. In contrast, the yields of 53BP1 and pKAP-1 foci were significantly higher than their corresponding background levels only when the cumulative dose reached 2.89 mGy (Fig. 2B), but they are more sensitive to radon exposure than their tracks. Collectively, these results show that the formation of γ-H2AX foci is more sensitive to radon exposure than the formation of 53BP1 and pKAP-1 foci.

The dose-response curves for radon exposure-induced γ-H2AX and pKAP-1 foci in HPBLs are shown in Fig. 2C,D. Over a dose range of 0–6.85 mGy, γ-H2AX and pKAP-1 foci exhibited linear dose-response relationships with cumulative absorbed doses from radon exposure. It is worth noting that there was no significant difference between the γ-H2AX foci dose-response curves when HPBLs were exposed to different concentrations of radon. Similar results were obtained for the dose-response curves of pKAP-1 foci, further indicating the relationships between foci response and radon exposure dose were determined by cumulative absorbed dose from radon exposure and were independent on radon concentration.

### HPBLs show sustained formation of γ-H2AX, 53BP1, pKAP-1 foci and their linear tracks after termination of radon exposure

Temporal changes in the yields of γ-H2AX, 53BP1 and pKAP-1 foci and their linear tracks in HPBLs were examined at various times after termination of exposure to radon at a dose of 5.56 mGy (Fig. 3). It was found that immediately after termination of radon exposure (0 h), as well as at 3 h and 6 h after termination of radon exposure, the yields of γ-H2AX, 53BP1 and pKAP-1 foci were drastically increased compared to the corresponding background levels with a peak at 3 h after termination of radon exposure (Fig. 3A). Moreover, the yield of γ-H2AX foci was much higher than those of 53BP1 and pKAP-1 foci within 6 h after termination of radon exposure and remained significantly higher than background value at 12 h after termination of radon exposure. In contrast, the number of residual 53BP1 and pKAP-1 foci had declined to near-background levels by 12 h (Fig. 3A). For linear foci tracks in HPBLs exposed to radon and its progeny, the majority of the linear γ-H2AX, 53BP1 and pKAP-1 foci tracks appeared intermittent at 0–12 h after termination of radon exposure except that the continuous linear γ-H2AX foci tracks were observed within the first 3 h after termination of radon exposure. The numbers of linear γ-H2AX, 53BP1 and pKAP-1 foci tracks at 0, 3 and 6 h after termination of radon exposure were significantly higher than their corresponding background levels with no significant differences between 0 h and 3 h and a decline from 3 h to 6 h, whereas the yield of linear γ-H2AX foci tracks was markedly higher than those of 53BP1 and pKAP-1 foci tracks within 6 h after termination of radon exposure (Fig. 3B). At 12 h after termination of radon exposure, the yields of all three types of linear foci tracks were markedly reduced compared to their corresponding yields at 0 h. However, the number of residual linear γ-H2AX foci tracks remained significantly higher than the background value. In contrast, the yields of 53BP1 and pKAP-1 foci tracks present at 12 h were not significantly different from the corresponding background levels. Together, these results demonstrate that the increased yields of γ-H2AX foci and its linear tracks in HPBLs are sustained for 12 h after termination of radon exposure.

### γ-H2AX, 53BP1 and pKAP-1 foci show an overdispersed contaminated Poisson distribution among HPBLs exposed to the background level and different doses of radon and its progeny

Poisson distribution analysis was performed to examine the distribution of γ-H2AX, 53BP1 and pKAP-1 foci among HPBLs exposed to the background level and different doses of radon. As for each type of foci, all the samples had μ values greater than |1.96| and δ^2^/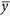 ratios greater than 1 showing an overdispersed contaminated Poisson distribution whether in the background control group or radon-exposed groups. Moreover, the distribution was independent of the concentration of radon and its progeny and of the cumulative absorbed dose from radon exposure (Table 1; 53BP1 foci- and pKAP-1 foci-related data not shown). Importantly, the number of foci formed in foci-positive cells increased gradually with the radon exposure doses. The majority of γ-H2AX foci-, 53BP1 foci- and pKAP-1 foci-positive cells contained only one focus in the blank control group and the radon-exposed groups with low doses, whereas the cells containing six γ-H2AX foci were only detected at a radon exposure dose of 5.51–5.80 mGy, and the cells containing four 53BP1 or pKAP-1 foci were detected only when the absorbed dose from radon exposure reached 5.80 mGy.

### Exposure to radon and its progeny induces the co-localization of the three types of foci (γ-H2AX, 53BP1 and pKAP-1) in HPBLs

**Figure 4:**
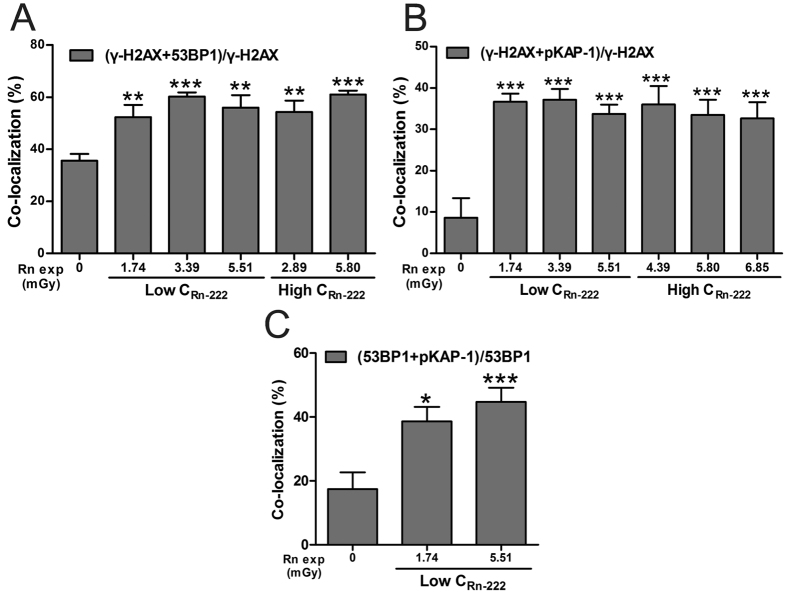
Ratios of γ-H2AX, 53BP1 and pKAP-1 foci co-localization in HPBLs *in vitro* exposed to radon and its progeny. (**A**) Co-localization of γ-H2AX foci and 53BP1 foci. (**B**) Co-localization of γ-H2AX foci and pKAP-1 foci. (**C**) Co-localization of 53BP1 foci and pKAP-1 foci. Two to four thousand lymphocytes from each sample were used for each pair foci quantitation. The data are presented as the mean ± standard deviation of three human blood samples. ^*^P < 0.05, ^**^P < 0.01, ^***^P < 0.001 compared with corresponding controls that were exposed to the natural background concentration of radon. Rn exp, radon exposure.

The co-localization of γ-H2AX/53BP1, γ-H2AX/pKAP-1 and 53BP1/pKAP-1 foci were observed in HPBLs after 1–6 h of *in vitro* radon exposure (Figs 1A and 2A). As shown in Fig. 4, the ratios of γ-H2AX/53BP1 foci co-localization, γ-H2AX/pKAP-1 foci co-localization and 53BP1/pKAP-1 foci co-localization were significantly higher in HPBLs that received various cumulative absorbed doses from radon exposure than in the blank control group. However, the co-localization ratios were not significant changed as the cumulative absorbed dose increased. In addition, the co-localization ratios were also independent of the concentration of radon and its progeny. pKAP-1 foci, a marker for heterochromatic DSBs, was co-localized with more either γ-H2AX or 53BP1 foci after radon exposure than the background control, indicating that γ-H2AX, 53BP1 and pKAP-1 participate in the repair of heterochromatic DSBs in G0 phase HPBLs. Our data support the notion that 53BP1, γ-H2AX and pKAP-1 jointly promote the repair of heterochromatic DSBs in the G1 phase^15^.

### Exposure to radon and its progeny induces the apoptotic pan-nuclear γ-H2AX response in HPBLs

**Figure 5:**
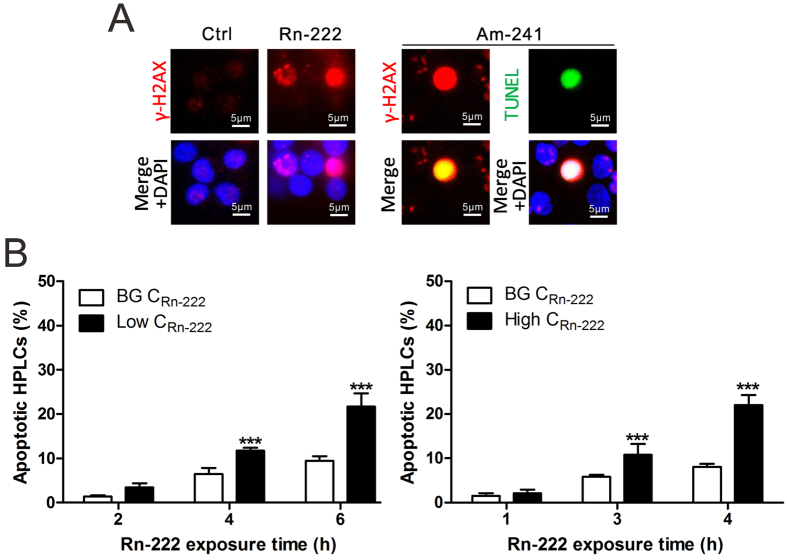
Apoptotic pan-nuclear γ-H2AX response in HPBLs *in vitro* exposed to radon and its progeny. (**A**) Representative images of pan-nuclear γ-H2AX staining in HPBLs induced by *in vitro* exposure to radon and its progeny and the co-localization of pan-nuclear γ-H2AX signal with TUNEL staining in HPBLs induced by α-particle irradiation from ^241^Am source at 6 h post-irradiation. (**B**) Relationship between the percentages of pan-nuclear γ-H2AX-positive cells and radon exposure time in HPBLs. One thousand HPBLs from each sample were used for quantitation. The data are presented as the mean ± standard deviation of three human blood samples. Red, γ-H2AX; Green, TUNEL staining; blue, DNA stained with DAPI. 1000 × magnification. ^***^P < 0.001 compared with the control group that was exposed to the natural background levels of radon. BG, background.

It has been reported that IR-induced pan-nuclear γ-H2AX response represents an apoptotic signal^33^,^34^,^35^,^36^. In the present study, we further observed that the pan-nuclear γ-H2AX response in HPBLs induced by α-particle irradiation from ^241^Am source was co-localized with apoptotic TUNEL staining accompanying with γ-H2AX foci in neighboring cells; exposure to radon also induced the apoptotic pan-nuclear γ-H2AX response accompanying with intermittent linear γ-H2AX foci track and individual foci in neighboring cells (Fig. 5A). The percentages of apoptotic HPBLs were significantly increased with radon exposure time for 3–6 h at 3.4–5.8 mGy cumulative dose with the maximum percentage of apoptotic HPBLs at about 22% (Fig. 5B), which is similar to the findings on the cell survival after 3 and 6 h radon exposure reported by other laboratory^37^. Notably, although the percentages of apoptotic HPBLs with radon exposure at natural environmental levels were increased with exposure time, they reached to merely 10% after 6 h-exposure, suggesting that *in vitro* radon exposure system is feasible.

## Discussion

In the present study, two patterns of linear γ-H2AX foci tracks, continuous and intermittent linear tracks, were observed in HPBLs during radon exposure. This finding is consistent with our recent report on *in vitro* exposure of rat PBLs to radon^31^. It is noteworthy that the formation of continuous linear γ-H2AX foci tracks was sustained within the first 3 h after termination of radon exposure, and that the formation of intermittent linear γ-H2AX foci tracks was sustained for 12 h after termination of radon exposure. Therefore, the formation and pattern of linear γ-H2AX foci tracks are not only useful in determining whether a person is suffering from a high dose of radon exposure, but also able to predict the period after a person with a high dose of radon exposure. This phenomenon may be explained by the dissolution of radon gas in cell culture medium and the resulting deposition of radon progeny, which results in sustained internal α-particle irradiation. This is also the important reason that yields of linear γ-H2AX, 53BP1 and pKAP-1 foci tracks remained constant within the first 3 h after termination of radon exposure and yields of their foci significantly increased within the first 6 h after termination of radon exposure compared to 0 h after termination of radon exposure.

The present study showed that the yields of radon and its progeny exposure-induced γ-H2AX, 53BP1 and pKAP-1 foci and their linear tracks in HPBLs are positively correlated with the cumulative absorbed dose and exhibit a linear dose-response relationship. This linear dose-response mode is consistent with the finding that high-LET radiation such as α particles, heavy ions and neutrons induces simple and complex chromosome aberrations as well as dicentric chromosome aberrations^38^,^39^. In the present study, we also found that the linear dose-response curves of γ-H2AX foci, pKAP-1 foci and their linear tracks showed no significant differences in HPBLs exposed to different concentrations of radon. This phenomenon is likely related to the fact that high-LET radiation lacks a dose-rate effect^40^. It is particularly noteworthy that the yields of γ-H2AX foci and its linear tracks were significantly higher than the background value in HPBLs exposed to extremely low dose of radon (1.74 mGy). In contrast, the yields of 53BP1 foci, pKAP-1 foci and their linear tracks in HPBLs that received a 1.74 mGy of radon did not differ significantly from the corresponding background values. These findings indicate that γ-H2AX foci and its linear track display greater sensitivity to irradiation than 53BP1 foci, pKAP-1 foci and their linear tracks. Moreover, at 12 h after termination of radon exposure, the levels of residual γ-H2AX foci and linear γ-H2AX foci tracks in HPBLs remained significantly higher than background values whereas the levels of residual 53BP1 foci, pKAP-1 foci and their linear tracks declined to near-background levels. We believed that the high yield of γ-H2AX foci induced by radon exposure, which is dependent on co-localization of γ-H2AX foci with 53BP1 or pKAP-1 foci, is the important reason for the significant increase of residual γ-H2AX foci level even at 12 h after termination of radon exposure. These results are consistent with the literature on that the yield of γ-H2AX foci induced by IR is significantly higher than those of 53BP1 foci and pKAP-1 foci^16^,^41^. Notably, our finding on the sustained increase of γ-H2AX level induced by radon exposure is supported by other studies. Liu *et al*. demonstrated that exposure to radon induced marked increases of γ-H2AX protein level and DNA damage detected by comet assay in human bronchial epidermal cells at 24 h after termination of radon exposure^42^. In addition, the epidemiological studies showed that the radon level in indoor air was positively associated with the level of DNA damage in PBLs of residents with indoor radon exposure^43^,^44^. Moreover, it was reported that the increased residual 53BP1/γ-H2AX foci remained in HPBLs for up to four weeks after γ-ray irradiation^20^. Our recent study also demonstrated that yields of γ-H2AX foci were significantly increased in rats inhaled 30 and 60 WLM radon for about 3 weeks and 5 weeks compared to the rats exposed to radon at background levels^31^. Importantly, the dose-response curves for γ-H2AX foci and its linear tracks in HPBLs *in vitro* exposed to radon and its progeny established in our present study were almost identical to those of rat PBLs *in vitro* exposed to radon and its progeny in our recent reports^31^, indicating that γ-H2AX foci-based assay to determine biological dose to PBLs and red bone marrow in rats inhaling radon and its progeny is feasible for human. Finally, immunofluorescence γ-H2AX staining has an advantage of showing apoptotic pan-nuclear γ-H2AX response to avoid the effects of apoptosis on residual foci counting^20^. Altogether, our results indicate that γ-H2AX foci and its linear tracks represent the best indicators for estimating the biological dose from extremely low-dose exposure to radon and its progeny and for determining whether a person has suffered high radon exposure.

As expected, the present study found that the distribution of γ-H2AX, 53BP1 and pKAP-1 foci induced by α-particle irradiation from radon and its progeny among HPBLs did not conform to the Poisson distribution and appeared to be overdispersed. Our finding is consistent with previous report that γ-H2AX foci induced by *in vitro* external high-LET neutron irradiation exhibited an overdispersed non-Poisson distribution in HPBLs^45^. It is noteworthy that the distribution of γ-H2AX foci in HPBLs *in vitro* irradiated by external low-LET γ-ray irradiation is also a non-Poisson distribution, showing an under discrete distribution at the radiation dose above 0.2 Gy and an overdispersed distribution at the radiation dose equal to or less than 0.1 Gy^45^. Together, these results therefore indicate that the distribution of γ-H2AX foci cannot be used to distinguish whether a person is exposed to low-LET radiation or high-LET radiation.

In summary, we demonstrated that the yields of γ-H2AX, pKAP-1 foci and their linear tracks displayed a linear dose-response relationship with the cumulative absorbed dose from radon exposure, and were independent of the radon concentration. Compared to 53BP1 foci, pKAP-1 foci and their linear tracks, γ-H2AX foci and its linear track are more sensitive indicators for determination of exposure to radon and its progeny with the lowest estimable dose of 1.74 mGy. Moreover, constant formation of γ-H2AX foci and its linear track was detected in HPBLs after termination of radon exposure, providing evidence for the feasibility of estimating the biological dose from internal α-particle irradiation. On the other hand, the γ-H2AX foci induced by exposure to radon and its progeny or by neuron and γ-ray irradiation exhibit an non-Poisson distribution in HPBLs, thus the γ-H2AX foci distribution cannot be employed to distinguish between low-LET and high-LET irradiation.

## Materials and Methods

### Collection of blood specimens and isolation and culture of HPBLs

A total of 6 healthy volunteers who had no history of exposure to radiation or contact with chemical toxic substances and were not addicted to tobacco or alcohol were selected for the present study. All the volunteers signed the informed consent documents. The research was approved by the Institutional Review Board of the Fudan University School of Public Health, and was conducted in accordance with the approved guidelines.

Peripheral blood was collected and placed in vacuum tubes containing the anticoagulant heparin. HPBLs were isolated by density gradient centrifugation in Ficoll-Paque (Cedarlane Co., Burlington, Ontario, Canada) according to the manufacturer’s instructions. The purity of HPBLs was at least 95% as determined by morphological criteria. The viability of cells from these separations was greater than 95% as determined by the trypan blue exclusion assay. Isolated HPBLs were cultured at a density of 1 × 10^6^ cell/ml in RPMI 1640 medium (Gibco®, Invitrogen Technologies, Carlsbad, CA, USA) supplemented with 15% fetal calf serum (Invitrogen Technologies), 5% (v/v) plasma, 100 U/ml penicillin, and 100 μg/ml streptomycin at 37 °C in a humidified incubator.

### *In vitro* exposure of HPBLs to radon and cumulative absorbed dose estimation

The *in vitro* radon exposure system has been described in our previous study^31^. Briefly, radon gas emanating from a ^226^Rn source was introduced at a chosen concentration in a closed chamber with saturated humidity placed in the water-jacketed incubator with constant temperature at 37 °C. The monolayers of spherical HPBLs attached in the permeable polyethylene terephthalate (PET) membrane of inserts were floated on the culture medium in the lower chamber of 6-well Transwell plates so that they can be irradiated by direct deposition of radon and its progeny from 0 ~ 180 degree any angle. Exposure of HPBLs to radon was achieved by placing the 6-well Transwell plates for 1–6 h in an *in vitro* radon exposure apparatus where the concentration of radon gas was continuously monitored with a RAD7 Professional Electronic Radon Detector (Durridage Company Inc., Billerica, MA, USA). For low radon exposure, radon gas coming from ^226^Rn source of activity 65 kBq was introduced at ~500 kBq/m^3^. For high radon exposure, radon gas coming from ^226^Rn source of activity 135 kBq was introduced at ~1000 kBq/m^3^. A solid-state CR-39 nuclear track detector (Fukuvi Chemical Industry Co., Ltd, Fukui, Japan) was simultaneously placed in a cell free insert of 6-well Transwell plate to measure the cumulative absorbed dose from radon exposure. The cumulative absorbed dose to the cells was calculated from the track density F (tracks/μm^2^) of the CR-39 sheet and the LET value (139 keV/μm) in our *in vitro* radon exposure system using our previous reported method^31^. The blank control group was simultaneously exposed to radon at the natural background level in another cell culture incubator with other conditions being the same. After radon exposure, the cells were collected, washed with phosphate-buffered saline (PBS), resuspended in PBS for cell-based immunofluorescence assays.

### Immunofluorescence assay

HPBLs suspensions at approximately 4 × 10^5^ cells/ml were deposit on glass slides in a monolayer by a cytocentrifuge using our previous reported method^31^. Subsequently, the cells were fixed in 4% paraformaldehyde/PBS for 15 min, permeabilized with 0.5% Triton X-100/PBS for 15 min and blocked with 10% calf serum/PBS for 1 h at 37 °C. After blocking, the cells were incubated with anti-γ-H2AX antibody (Cell Signaling Technology, Inc., Danvers, MA, USA; 1:500 dilution), anti-pKAP-1 (Ser824) antibody (Bioss Inc., Woburn, MA, USA; 1:500 dilution) or anti-53BP1 antibody (R&D Systems Inc., Minneapolis, MN, USA; 1:200 dilution) at 4 °C overnight and then incubated with the corresponding fluorescent secondary antibodies, Alexa Fluor 488 or Alexa Fluor 555 (Molecular Probes, Life Technologies, Grand Island, NY, USA) for 1 h at room temperature (RT) in the dark. The cells were stained in anti**-**fade mounting medium containing 4′,6-diamidino-2-phenylindole (DAPI, Santa Cruz Biotechnology, Inc., Santa Cruz, CA, USA), covered with cover slips, sealed with clear nail polish and examined under a fluorescence microscope.

### Fluorescence microscopic examination and criteria for counting foci and their tracks

The scoring criteria for counting individual foci and continuous and intermittent linear foci tracks has been described in our previous report^31^. A linear track containing more than three isolated foci along a straight line is defined to an intermittent linear track, and isolated foci on a intermittent linear track is counted as individual foci together with dispersed individual foci. Using an Olympus BX51 fluorescence microscope (Tokyo, Japan), the number of γ-H2AX foci and its linear tracks, 53BP1 foci and its linear tracks, and pKAP-1 foci and its linear tracks were determined and recorded per 2000–4000 nuclei in each sample. The co-localization status of γ-H2AX foci and 53BP1 foci, γ-H2AX foci and pKAP-1 foci, or 53BP1 foci and pKAP-1 foci was also determined and recorded. Co-localization with γ-H2AX foci was calculated as the ratio of the number of co-localized γ-H2AX/53BP1 foci to the total number of γ-H2AX foci and the ratio of the number of co-localized γ-H2AX/pKAP-1 foci to the total number of γ-H2AX foci. Similarly, co-localization with 53BP1 foci was calculated as the ratio of the number of co-localized 53BP1/pKAP-1 foci to the total number of 53BP1 foci.

### Statistical analysis

All data are expressed as 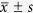. The data were analyzed using SPSS 17.0 software. Student’s t-test was conducted to compare data between two groups; one-way analysis of variance (ANOVA) was employed to compare data among multiple groups. P values less than 0.05 were considered statistically significant.

The distribution of γ-H2AX foci, pKAP-1 foci and 53BP1 foci in the HPBLs was subjected to Poisson distribution analysis (μ test) using Ctampois software, a generous gift from Professor Lloyd DC (Cytogenetics Laboratory, National Radiological Protection Board, UK). The μ value and the ratio of variance to the mean (δ^2^/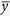) were calculated. A μ value less than |1.96| and a δ^2^/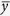 ratio close to 1.00 indicated that the foci distribution conformed to a Poisson distribution. In contrast, foci distribution did not meet the assumptions of the Poisson distribution if the μ value was greater than |1.96| and the δ^2^/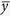 ratio was not close to 1.00. Moreover, μ > |1.96| and δ^2^/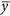 < 1.00 indicated lack of discrete distribution, whereas μ > |1.96| and δ^2^/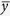 > 1.00 indicated overdispersion.

## Additional Information

**How to cite this article**: Ding, D. *et al*. γ-H2AX/53BP1/pKAP-1 foci and their linear tracks induced by *in vitro* exposure to radon and its progeny in human peripheral blood lymphocytes. *Sci. Rep.* **6**, 38295; doi: 10.1038/srep38295 (2016).

**Publisher's note:** Springer Nature remains neutral with regard to jurisdictional claims in published maps and institutional affiliations.

## Figures and Tables

**Table 1:** Distribution of γ-H2AX foci in HPBLs exposed to radon and its progeny.

		No. of samples	Total cell count	γ-H2AX foci/cell	Cells containing different number of γ-H2AX foci							δ^2^/ 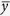	μ
Groups					0	1	2	3	4	5	6		
**Ctrl**	0 mGy	3	4006	0.009	3980	16	8	2	0	0	0	1.728	23.67
			4024	0.013	3988	26	6	2	2	0	0	1.911	29.46
			4010	0.011	3978	24	4	2	2	0	0	1.946	30.62
		mean ± SD	4013 ± 9	0.011 ± 0.002	3982 ± 5	22 ± 5	6 ± 2	2 ± 0	1 ± 1	0	0	1.989	32.00
**Low C**_**Rn-222**_	1.74 mGy	3	4057	0.024	3979	67	4	6	1	0	0	1.554	25.07
			4071	0.023	4000	54	10	7	0	0	0	1.630	28.56
			4081	0.021	4017	47	13	4	0	0	0	1.568	25.80
		mean ± SD	4070 ± 12	0.023 ± 0.002	3999 ± 19	56 ± 10	9 ± 5	6 ± 2	0 ± 1	0	0	1.565	25.61
	3.39 mGy	3	4084	0.036	3970	94	6	14	0	0	0	1.613	27.78
			4208	0.033	4108	77	9	13	0	1	0	1.802	36.91
			4062	0.036	3948	90	15	9	0	0	0	1.536	24.22
		mean ± SD	4118 ± 79	0.035 ± 0.002	4009 ± 87	87 ± 9	10 ± 5	12 ± 3	0	0 ± 1	0	1.609	27.73
	5.51mGy	3	4065	0.062	3910	94	35	19	4	2	1	2.132	51.13
			4002	0.065	3852	89	28	23	6	3	1	2.310	58.72
			4043	0.061	3897	87	34	17	3	3	2	2.271	57.24
		mean ± SD	4037 ± 32	0.063 ± 0.002	3886 ± 30	90 ± 4	32 ± 4	20 ± 3	4 ± 2	3 ± 1	1 ± 1	2.221	54.96
**High C**_**Rn-222**_	2.89 mGy	3	4006	0.035	3924	44	22	10	6	0	0	2.205	38.40
			4084	0.035	4004	42	22	8	6	2	0	2.383	44.48
			4000	0.033	3924	42	20	8	6	0	0	2.199	38.21
		mean ± SD	4030 ± 47	0.034 ± 0.002	3951 ± 46	43 ± 1	21 ± 1	9 ± 1	6 ± 0	1 ± 1	0	2.192	38.09
	5.80 mGy	3	4008	0.063	3866	78	32	22	6	2	2	2.387	44.06
			4000	0.063	3856	86	30	18	2	4	4	2.494	47.41
			4000	0.062	3852	92	30	16	4	4	2	2.326	42.10
		mean ± SD	4003 ± 5	0.063 ± 0.001	3858 ± 7	85 ± 7	31 ± 1	19 ± 3	4 ± 2	3 ± 1	3 ± 1	2.375	43.65

Abbreviation: SD, standard deviation.
